# “For me, it's connection”: Older Adults Experience with Technology during COVID-19

**DOI:** 10.1093/geroni/igab046.3550

**Published:** 2021-12-17

**Authors:** Vivian Miller, HeeSoon Lee, Erin Roark

**Affiliations:** 1 Bowling Green State University, Bowling Green, Ohio, United States; 2 Augusta University, Augusta, Georgia, United States

## Abstract

As a result of COVID-19, older adults have experienced isolation, lost social contacts, and a decrease in connections. A recent study found that “approximately one-quarter of community-dwelling older adults are considered to be socially isolated, and 43% of them report feeling lonely.” Various innovative interventions have emerged, including technology-based interventions as a means to reduce social isolation in older adults, particularly as information communication technology (ICT) use is on the rise among this population. However, it remains to be known how these connections are faring for older adults in the pandemic and whether these ICT connections lead to greater or lesser feelings of social connectedness. Thirty-nine (N=39) in-depth semi-structured interviews were conducted to explore the lived experiences of technology use among older adults during COVID-19. Participants experiences with ICT ranged from illiterate to savvy. Most participants indicated Zoom was the primary means to stay socially connected to family and friends. Participants emphasized that ICT may be a possible solution to deal with loneliness for those older adults who are especially isolated due to COVID-19 restrictions. Barriers and challenges to ICT use included taking too much time to use and needing help to fix any problems that arose. Finally, participants shared essential aspects of ICT use, revealing that it was ‘technology or nothing.’ Findings from this study indicate a need for a simple ICT for the older adult population. Moreover, findings suggest opportunities for peer-support ICT training programs for older adults.

